# A Social-Ecological Approach to Understanding Adolescent Sexting Behavior

**DOI:** 10.1007/s10508-021-01988-9

**Published:** 2021-05-12

**Authors:** Simon C. Hunter, Kirsten Russell, Stefania Pagani, Lindsey Munro, Sofia M. Pimenta, Inmaculada Marín-López, Jun Sung Hong, Lee Knifton

**Affiliations:** 1 Department of Psychology, Glasgow Caledonian University, Glasgow, G4 0BA UK; 2 Faculty of Education, University of Western Australia, Perth, Australia; 3 School of Psychological Sciences and Health, University of Strathclyde, Glasgow, UK; 4 Department of Education, University of Córdoba, Córdoba, Spain; 5 Department of Adult Health, School of Social Work, Wayne State University, Detroit, MI USA; 6 Dept of Management Science, University of Strathclyde, Glasgow, UK

**Keywords:** Sexting, School connectedness, Parenting, Peer-pressure, Romantic-pressure

## Abstract

This study examined the extent to which active and passive sexting behaviors are associated with family-, school-, peer-, and romantic-level variables. Young people (*N* = 3,322; 49.1% female, 48.3% male, 2.6% other) aged 11 to 15 years old (*M* = 12.84, *SD* = 0.89) took part, and all attended mainstream secondary schools in Scotland. Participants completed self-report measures of school connectedness, parental love and support, perceived susceptibility to peer- and romantic-pressure (e.g., to display behaviors just to impress others), and their involvement in active and passive sexting. The importance of both school- and family-level factors was evident, though perceived romantic-pressure had the largest effect. However, neither school- nor family-level variables were moderated by either perceived romantic-pressure or perceived peer-pressure. Efforts to reduce sexting or increase its safety should primarily seek to tackle young people’s ability to respond effectively to romantic-pressure. It may also be helpful to develop school connectedness and to help families provide support that is constructive and not intrusive.

## Introduction

Sexting refers to sending or receiving of sexually explicit messages or images using the internet or mobile phone (Choi et al., 2016). Adolescents often see sexting as normal behavior (Lippman & Campbell, 2014; Walker et al., 2013) despite the legal (Lee & Darcy, 2021; O'Connor et al., 2017), social (Lippman & Campbell, 2014; Strassberg et al., 2013), and interpersonal (Van Ouytsel et al., 2017) problems that are associated with it. To better understand this behavior, it is important to study it within a social-ecological framework that acknowledges the influence of factors at various levels. As outlined below, the decision to engage in sexting is likely to be influenced by competing factors, including variables at the individual, peer, family, and school levels. In this study, we examine the extent to which both active sexting (sending or requesting sexts) and passive sexting (receiving or being asked for sexts) are associated with variables at each level of the social-ecological framework, and whether family- and school-level factors can reduce the negative outcomes associated with peer and romantic interactions.

### Sexting

Sexting is an active behavior involving actions such as asking others to send sexts, sending sexts oneself, or forwarding on sexts. It includes a complex set of behaviors and experiences which differ in the degree to which they are potentially risky. Sexting can also be a passive experience where a young person is the target of sexting behavior, such as, being asked to sext or having unsolicited sexts sent or forwarded to them (Ojeda et al., 2019).

Recent meta-analyses indicate that 14.8% of young people (under 18 years old) report sending a sext, 27.4% have received a sext, and 8.4% have forwarded a sext without consent (Madigan et al., 2018). Among young adults, aged 18–28, this increases to 38.3% who send a sex, 41.5% who receive a sext, and 47.7% who engage in reciprocal sexting (Mori et al., 2020). While sexting is associated with both positive and negative emotions (Mitchell et al., 2012; Villacampa, 2017), there is compelling evidence that sexting can be a problematic experience for young people in terms of its associations with risky sexual behavior (e.g., failing to use contraception, having multiple sex partners), delinquent behavior, anxiety/depression, and substance abuse (Mori et al., 2019).

### Social-Ecological Framework and Sexting in Young People

Bronfenbrenner’s (1979, 1994) ecological systems framework proposes that individual behavior, such as sexting, is influenced by “the ecological environment [which] is conceived as a set of nested structures, each inside the next…” (Bronfenbrenner, 1979, p. 3). The individual is considered to be an inseparable part of multiple, interrelated systems, including the microsystem (i.e., immediate settings such as home or school, e.g., peer relations), mesosystem (i.e., the interrelations among the microsystems, e.g., the relationship between peers and romantic partners), exosystem (i.e., settings which can influence the individual though they are not directly involved in them, such as a parent’s workplace), and macrosystem (i.e., cultural or subcultural patterns).

The current study focuses on the microsystem and the mesosystem. These include individual-, peer-, family-, and school-level variables (microsystem), which we will discuss first below. Subsequently, we will also discuss potential interactions between these (mesosystem). Issues relating to peer-pressure are particularly germane for young people since adolescence is a developmental period characterized by increasing independence from adults, allied with the dangers of powerful peer-socialization effects, which are often evident in risky behaviors (Dumas et al., 2012; Kiesner et al., 2002). Young people, particularly males, may pressure their romantic partners into sexting behavior (Lippman & Campbell, 2014; Martinez-Prather & Vandiver, 2014; Van Ouytsel et al., 2017; Walker et al., 2013), and pressure from peers more generally can also be associated with more sexting (Lee et al., 2016; Walrave et al., 2014).

Beyond these effects, the extent to which young people identify or connect with their school may also be associated with less sexting. School connectedness is associated with less frequent reports of other risky sexual behaviors (Aspy et al., 2012; Handebo et al., 2018; Shneyderman, & Schwartz, 2013) as well as lower rates of high-risk or delinquent behaviors, such as smoking and alcohol consumption (Aspy et al., 2012; Azagba & Asbridge, 2013; Bond et al., 2007; Resnick et al., 1997). Walrave et al. (2014) found that the degree to which young people believe their teachers would approve of them sending sexts, and whether they attached any importance to their teachers’ opinions of sexting, did not predict sexting intention among young people. However, to date, no study has examined whether school connectedness is associated with self-reports of sexting behaviors and experiences.

A second contextual school-level variable is the extent to which a school explicitly includes education and support for students surrounding positive interactions. Many U.S. and UK schools are tackling such issues via the Mentors in Violence Prevention (MVP) intervention (Katz, 1995). The MVP program encourages young people to intervene, nonviolently, when they witness violence and challenges their norms around gender-based violence as they work through a “playbook” of scenarios (including one scenario on sharing sexual images of peers). We expect that MVP may reduce sexting rates, predicated upon an understanding of sexting that explicitly includes the abuse of power in a romantic relationship. This is a contentious assertion, given that consenting adults can find sexting to be an enjoyable and positive experience (Burkett, 2015). However, among minors, sexting is usually an illegal activity (O'Connor et al., 2017), and young people, especially girls, can often feel pressured to participate (Lee et al., 2016; Lippman & Campbell, 2014; Walrave et al., 2014), which suggests that it is often abusive and selfish behavior. In this way, we expect that young people attending schools that participated in the MVP intervention would report lower levels of sexting.

The family is also a salient microsystem for young people, and important attitudes and behaviors are formed in the home, which can then impact the interactions outside of that context (Bronfenbrenner, 1989). Caregivers have their own opinions about sexting and their own ideas about how to tackle it (Fix et al., 2021). Some familial variables are not related to young people’s reported intentions to sext, including family living arrangements (Van Ouytsel et al., 2014), parental education (Villacampa, 2017), and parental opinions of sexting (Walrave et al., 2014). However, young people who do not sext do report being more concerned about the consequences of being caught sexting by their parents (Lippman & Campbell, 2014). Studies to date have not directly considered how the quality of parent–child relationship relates to sexting, yet feeling cared for and loved by one’s parents is critical for positive development and psychological adjustment (Sabey et al., 2018) and may be important for this behavior (Lippman & Campbell, 2014).

In addition to microsystems, it is of theoretical and practical importance to also consider the mesosystem, that is, the ways in which romantic-pressure, peer-pressure, school connectedness, and perceived parental love and support may interact and influence each other. This is an important theoretically-informed aspect to study if we want to understand and describe the ways that these operate in young people’s lives. In a separate field of research (bullying), scholars have reported interactions between family- and peer-level variables concerning cigarette use, such that higher parental monitoring buffered young people from smoking when they also experienced bullying (Lee et al., 2018). Research also shows that a sense of community can protect young people whose peer and parental influences place them at risk for substance abuse (Mayberry et al., 2009) and that neighborhood socioeconomic status interacts with parenting practices when explaining the likelihood of a young person reporting having had sex (Roche et al., 2005). In the context of sexting, we propose that peer-level variables are likely to moderate any beneficial effects of school- and family-level variables. This is because adolescence is a developmental period characterized by increasing independence from adults and the effects of peer-socialization are evident in risky behaviors (Dumas et al., 2012; Kiesner et al., 2002).

Finally, with respect to the issue of active and passive sexting, these may be differentially associated with the microsystem variables. For example, perceived susceptibility to peer-pressure and romantic-pressure, perceived parental love and support, and school connectedness are all likely to be associated with actively sexting. We propose that all four variables will be associated with passive sexting because they are likely to be indicators of the extent to which young people end up in risky situations or risky peer groups where receiving sexts may be more likely. This is based on the assumption that young people who are susceptible to peer-pressure are more likely to belong to deviant or delinquent peer groups (Connolly et al., 2015; Farrell et al., 2017). Similarly, school-level variables and parenting practices can be important determinants of antisocial behavior (Higuita-Gutiérrez & Cardona-Arias, 2017; Kramer-Kuhn & Farrell, 2016; Patterson et al., 1990), and while sexting may be a normative and positive interaction, it also has the potential to be negative and to be associated with more abusive and antisocial behavior (Ojeda et al., 2019).

### Research Questions and Hypotheses

Here, we draw on Bronfenbrenner’s (1979, 1994) ecological systems framework to assess the relative contributions of peer-pressure, romantic-pressure, school connectedness, and parental love and support in adolescent sexting behavior, an important contribution if we aim to understand young people’s behaviors and experiences. Our hypotheses, preregistered on the Open Science Framework (OSF: see Hunter et al., 2019) are:^1^

Hypothesis 1a: School connectedness and parental love and support will both be negatively associated with involvement in active sexting (sending and/or asking for sexts), while perceived peer-pressure and perceived romantic-pressure will both be positively associated with involvement in active sexting.

Hypothesis 1b: Perceived peer-pressure and perceived romantic-pressure will moderate the associations between school connectedness and parental love and support and involvement in active sexting (sending and/or asking for sexts). Specifically, the effects of school connectedness and parental love and support will be weaker when perceived peer-pressure and perceived romantic-pressure are high.

Hypothesis 2: Compared to young people in non-MVP schools, young people attending MVP schools will report less active sexting and less passive sexting.

In addition to these proposed hypotheses, a more exploratory aim was to evaluate Hypotheses 1a and 1b for passive sexting. Finally, following peer review, all analyses were repeated omitting perceived romantic-pressure; this is therefore conducted as an additional exploratory analysis.

## Method

### Participants

A total of 3,322 young people attending 15 mainstream secondary schools in Scotland took part in the survey between Fall 2017 and Spring 2018. The mean age of the participants was 12.84 years (*SD* = 0.89, range 11–15), of whom 1,631 (49.1%) reported their sex as female, 1,606 (48.3%) as male, and 85 (2.6%) who did not report their sex. The sample predominantly identified as White (*n* = 3,116; 93.8%), and the remainder identified as Asian, Asian Scottish, or Asian British (*n* = 58; 2.6%), mixed or multiple ethnicity (*n* = 32; 1.4%), or another ethnic group. Of these, 55% were from schools participating in MVP, and 45% were from non-MVP schools. Across Scotland in 2017, 14.1% of the secondary school students were registered for free school meals (National Statistics, 2017). The schools participating in the current study had a range of 8.9% to 36.8% of students eligible for free school meals (*M* = 17.6%, *SD* = 7.02). The sample size exceeded the preregistered target sample size (*N* = 500: Hunter et al., 2019) because of schools’ enthusiasm and motivation to take part.

### Measures

#### Ethnicity

Six response options truncated from the Scottish Government’s census categories assessed ethnicity: “White,” “Asian, Asian Scottish, or Asian British,” “mixed or multiple ethnic group,” “African,” “Caribbean or Black,” and “Other ethnic group.” These categories were subsequently collapsed into White and non-White for the analysis due to small subsample sizes across each category (ranging from 4 to 58 youths).

#### Involvement in Sexting

Choi et al.’s (2016) four-item scale assessing involvement in sexting was used to explore what has the potential to be high-risk sexting behavior, namely images rather than text-only. Participants were asked to respond to the items with reference to “this school year” and responses were either “Yes” or “No.” Two scores were created for each participant. The first score represented reports of active sexting behavior and was scaled as 0, 1, or 2. This reflects whether the young person responded positively to neither, one, or both active sexting items (“Have you asked someone to send naked pictures of them to you?”; “Have you sent naked pictures of yourself to another through text, email, or SnapChat?”). The second score reflected passive sexting experiences and related to the items, “Have you been asked to send naked pictures of yourself through text, email, or things like SnapChat?” and “Has anyone sent you a naked picture without you asking?.”

#### School Connectedness

The 4-item school connectedness subscale of the Perceived School Experiences Scale (Anderson-Butcher et al., 2012) was used (e.g., “I feel like I belong to my school”). Possible responses were 1 = “strongly disagree,” 2 = “disagree,” 3 = “neither agree nor disagree,” 4 = “agree,” and 5 = “strongly agree.” A mean score was created from the responses to these items where higher scores indicated greater school connectedness. Internal reliability for this scale was satisfactory (α = 0.79).

### Parental Love and Support

Parental love and support was assessed with a 6-item scale used by Merrin et al. (2015). This scale utilized a 4-point scale (0 = “Never” to 3 = “Always”). An example item is “My parents/guardians…encourage me to do well.” A mean score was created, and higher scores reflect higher parental love and support. Internal reliability for this scale was satisfactory (α = 0.75).

#### Perceived Susceptibility to Peer-Pressure

Perceived susceptibility to peer-pressure was measured using the four items in Williams and Anthony’s (2015) study. An example item is “I tend to go along with the crowd,” and the response options were “Not like me,” “A little like me,” or “A lot like me.” Responses were scored so that a higher score reflected higher perceived susceptibility to peer-pressure. A mean score was created from responses to these items. Internal reliability for this scale was adequate (α = 0.63).

#### Perceived Susceptibility to Romantic-Pressure

Perceived susceptibility to romantic-pressure was assessed using an adapted version of Williams and Anthony’s (2015) scale. This measure was adapted to refer specifically to the romantic relationship context: “I do things just to be popular with my partner”; “I let my partner talk me into doing things I don’t really want to do”; “I try hard to impress my partner”; and “I tend to go along with the things my partner wants to do.” Response options were “Not like me,” “A little like me,” and “A lot like me,” which were scored in the same way as for peer-pressure. A mean score was used where higher scores indicated greater perceived susceptibility to romantic-pressure. Internal reliability for this scale was satisfactory (α = 0.74).

### Procedure

Ethical approval for the project was obtained from the second author’s institution. In Scotland, young people aged 12 and over can give their own consent to participate in if they are considered able to do so (e.g., not having a specific vulnerability such as mental or physical illness). However, given the potentially controversial nature of the questions included in this study (regarding sexting), we gave parents the opportunity to withdraw their child from participation. Approval was obtained from seven Local Education Authorities to contact schools to request their participation. Of those schools that were approached, 15 (32%) agreed to take part. This is consistent with previous research taking place in secondary schools in Scotland (see O’Connor et al., 2009; Russell et al., 2018). Schools were targeted based on their MVP status (engaged or not engaged with MVP).

The aim of the study was explained to school gatekeepers (e.g., Principals). Parents were informed of the project by letter and asked to notify the school or research team if they did not want their child to participate. Young people were then invited to participate in the study. Respondents completed the self-report surveys anonymously using paper-based copies (to allow, for example, covering of answers) and completed these independently within a classroom or assembly hall setting. Teachers and members of the research team made themselves available at all times to answer any questions that the students had regarding the survey procedure or content. All participants were debriefed and provided with information as to where they could seek support if they were concerned about bullying or sexting. All letters and information sheets used in the project are openly available in the preregistration for this project (Hunter et al., 2019).

### Analytic Plan

The data were analyzed using Mplus Version 7.31 (Muthén & Muthén, 1998–2012). Mplus allows missing data to be addressed using Full Information Maximum Likelihood (FIML). All main analyses were based on a subsample of the data (*n* = 2,253): The sex of the participants was treated as a binary variable with all respondents who chose “Prefer not to say” excluded due to the low frequency of such responses (*n* = 85). Additionally, survey instructions required participants to leave the perceived susceptibility to romantic-pressure items blank if they had never had a boyfriend or girlfriend, and 984 participants who did so were also therefore excluded. The Cronbach’s alphas for scale reliabilities reported above were calculated on this subsample.

The analyses differed slightly from the preregistration for this study. There was a clear repetition of the analyses across models and so a slightly more efficient analytic approach was used. Specifically, the effect of MVP/non-MVP school status on active and passive sexting was originally proposed to be assessed in one model (see the preregistered analysis for Hypothesis 2), and a separate model was planned to assess the effects of other study variables on active sexting (see the preregistered analysis for Hypothesis 1a). The evaluation of the MVP/non-MVP distinction was therefore included in the same analysis that assessed Hypothesis 1 so that its effects could be examined in the same model as other effects. In addition, the examination of the effects of study variables on passive sexting was not preregistered. Since active sexting and passive sexting were significantly correlated [*r* (df = 2251) = 0.34, *p* < .001], both forms of sexting were included as outcome variables in the same models rather than being estimated separately. Finally, when estimating the models assessing the possibility of the interactions, the MLR estimator was not used because bootstrapping was employed (precluding the use of the MLR estimator). Instead, the ML estimator is used, and we note that bootstrapping procedures also address non-normality in data.

Thus, we ran one analysis where both forms of sexting were regressed on school connectedness, parental love and support, perceived peer-pressure, perceived romantic-pressure, and MVP/Non-MVP status. Covariates were age, sex, and ethnicity. Descriptive statistics were calculated using SPSS version 25. Following this, we evaluated one model per possible interaction: (a) Perceived susceptibility to peer-pressure × School connectedness; (b) Perceived susceptibility to peer-pressure × Parental love and support; (c) perceived susceptibility to romantic-pressure × School connectedness; (d) perceived susceptibility to romantic-pressure × Parental love and support). Thus, four models were estimated in total to assess the interaction terms. This approach (conducting four separate analyses) was preregistered as the inclusion of multiple interaction terms sharing the same variables would be highly likely to result in difficulties with multicollinearity. The variables included in each interaction (e.g., perceived susceptibility to peer-pressure × school connectedness) were also included as predictors, and the two variables (e.g., perceived susceptibility to romantic-pressure and parental love and support) not contained in the interaction were included as covariates. The variables included in each interaction were mean-centered. In each model, covariates also included age, sex, ethnicity, and MVP/non-MVP status. Given the number of estimated parameters across all models, alpha was set at a 1% level rather than 5%. All code is hosted on the Open Science Framework (Hunter et al., 2019).

To explore significant interactions, it was preregistered that moderation procedures be implemented in Mplus (based on the code produced by Stride et al., 2015). However, no interaction terms were significant (all *p* > .107), and therefore, this was not required.

The main analyses were all repeated omitting perceived susceptibility to romantic-pressure. This was an exploratory set of analyses, and since they did not require that young people be in romantic relationships, we included the full data set.

## Results

Descriptive statistics are shown in Table 1. Active sexting was significantly correlated with all main study variables. The correlation with MVP status was small and was only significant at the 5% level. Passive sexting was significantly correlated with all main study variables except MVP status. The four main study variables were all significantly correlated with each other, most notably, school connectedness and parental love and support (*r*[2181] = 0.35, *p* < .001) and both forms of perceived pressure (*r*[2235] = 0.49, *p* < .001).

**Table 1:** Descriptive statistics for, and correlations between, all main study variables and covariates

Variable	2	3	4	5	6	7	8	9	10	*M* (SD) or %	Range
1. Active sexting	.34***	.15***	− .01	− .02	.05*	− .11***	− .12***	.11***	.16***	0.08 (0.33)	0–2
2. Passive sexting	–	.21***	.21***	− .03	.03	− .19***	− .15***	.12***	.18***	0.51 (0.78)	0–2
3. Age	–	–	− .02	.01	.05*	− .11***	− .04*	− .01	.06**	12.89 (0.89)	11–15
4. Sex^a^	–	–	–	− .07**	− .00	.08***	.08**	.12***	− .20***	51.0% Male	–
5. Ethnicity^b^	–	–	–	–	− .01	.00	− .03	.04	.03	95.0% White	–
6. MVP status^c^	–	–	–	–	–	.03	.06**	.00	− .01	56.9% MVP	–
7. SC	–	–	–	–	–	–	.35***	− .11***	− .10***	3.52 (0.77)	0.5–5
8. PLS	–	–	–	–	–	–	–	− .12***	− .10***	2.28 (0.56)	0–4
9. PPP	–	–	–	–	–	–	–	–	.49***	0.46 (0.42)	0–2
10. PRP	–	–	–	–	–	–	–	–	–	0.32 (0.41)	0–2

*Note*. SC = school connectedness; PLS = parental love and support; PPP = perceived susceptibility to peer-pressure; PRP = perceived susceptibility to romantic-pressure. Due to missing data, sample sizes across analyses ranged from 2,207 to 2,253

^a^Sex is coded as 1 = Boy, 2 = Girl

^b^Ethnicity is coded as 1 = White, 2 = Not White

^c^MVP status is coded as 0 = Non-MVP, 1 = MVP

*p < .05. **p < .01. ***p < .001

The first analysis, involving no interaction terms, accounted for 18% of the variance in passive sexting and 6% of the variance in active sexting (*R*^*2*^ = 0.180 and 0.064, respectively). The results are summarized in Table 2.

**Table 2:** Model results predicting active and passive sexting

Variable	Passive sexting				Active sexting			
	b (SE)	95%CI	Sig	*β*^*1*^	b (SE)	95%CI	Sig	*β*^*a*^
Age	0.17 (0.02)	0.13, 0.20	< .001	.19	0.05 (0.01)	0.03, 0.07	< .001	.13
Sex^b^	0.43 (0.03)	0.37, 0.49	< .001	.28	0.03 (0.01)	− 0.00, 0.05	.070	.04
Ethnicity^c^	− 0.05 (0.07)	− 0.18, 0.08	.418	− .02	− 0.03 (0.02)	− 0.07, 0.02	.197	− .02
MVP status^d^	0.05 (0.03)	− 0.01, 0.11	.087	.03	0.04 (0.01)	0.01, 0.06	.009	.05
SC	− 0.14 (0.02)	− 0.18, − 0.10	< .001	− .14	− 0.02 (0.01)	− 0.04, − 0.00	.040	− .05
PLS	− 0.13 (0.03)	− 0.19, − 0.07	< .001	− .09	− 0.05 (0.02)	− 0.08, − 0.02	.003	− .08
PPP	0.07 (0.04)	− 0.01, 0.15	.096	.04	0.03 (0.02)	− 0.01, 0.08	.206	.04
PRP	0.35 (0.05)	0.27, 0.43	< .001	.19	0.11 (0.03)	0.06, 0.16	< .001	.13
SCxPPP	0.06 (0.04)	− − 0.02, 0.14	.188	.03	0.05 (0.03)	− 0.01, 0.11	.107	.05
SCxPRP	− 0.05 (0.04)	− 0.13, 0.03	.266	− .02	0.01 (0.03)	− 0.05, 0.08	.672	.02
PLSxPPP	− 0.04 (0.06)	− 0.16, 0.09	.563	− .01	0.03 (0.05)	− 0.06, 0.12	.537	.02
PLSxPRP	− 0.02 (0.06)	− 0.15, 0.08	.564	− .01	0.02 (0.05)	− 0.06, 0.11	.606	.02

*Note*. SC = school connectedness; PLS = parental love and support; PPP = perceived susceptibility to peer-pressure; PRP = perceived susceptibility to romantic-pressure

^a^STDYX standardized results are reported

^b^Sex is coded as 1 = Boy, 2 = Girl

^c^Ethnicity is coded as 1 = White, 2 = Not White

^d^MVP status coded 0 = Non-MVP, 1 = MVP

Ethnic group status was not associated with sexting. Girls reported more passive sexting than boys (41.8% vs 24.3%) but did not differ on reports of active sexting. Age was significantly and positively associated with both forms of sexting. Active sexting was uncommon among 11- and 12-year-olds (1.1% and 1.8%, respectively) but became more frequent among 13- and 14-year-olds (5.6% and 11.1%, respectively). Passive sexting experiences also became steadily more frequent as the participants got older, as reported by 16.7% of the 11-year-olds, 20.6% of 12-year-olds, 36.4% of 13-year-olds, and 45.3% of 14-year-olds.

We found mixed support for Hypothesis 1a. School connectedness was significantly and negatively associated with passive sexting but was not associated with active sexting. This was the only difference between the two forms of sexting when considering the four main study variables, and it was contrary to our expectation that effects would be stronger for active than for passive sexting. Parental love and support was negatively associated with both passive sexting and active sexting. Perceived susceptibility to peer-pressure was not associated with either form of sexting, but perceived susceptibility to romantic-pressure was positively associated with reports of both passive sexting and active sexting. Finally, active sexting was higher among young people attending MVP schools than those not attending MVP schools, but this was not the case for passive sexting. Young people in MVP schools reported a higher prevalence of active sexting (6.9%) than those not in MVP schools (4.2%), contradicting Hypothesis 2.

Finally, none of the four interactions were significant (all *p*s ≥ 0.150) (see Table 2), thus failing to support Hypothesis 1b. This indicates that the school- and parent-level variables did not buffer young people against any negative effects of perceived peer- or romantic-pressure.

### Exploratory Analyses

When running the models without including perceived susceptibility to romantic-pressure (and hence including *all* participants), there were two main differences. First, in the model, which did not include the interaction terms, the pattern of results was largely similar in terms of significance and sizes of effects except that (a) MVP and non-MVP schools did not differ on active sexting (b = 0.02, SE = 0.01, *p* = .046, *β* = 0.03), and (b) perceived susceptibility to peer-pressure was a significant predictor of both active (b = 0.07, SE = 0.02, *p* < .001, *β* = 0.10) and passive (b = 0.23, SE = 0.03, *p* < .001, *β* = 0.13) sexting.

The interaction term perceived susceptibility to peer-pressure × school connectedness was not significant for either active (b = 0.03, SE = 0.02, *p* = .253, *β* = 0.03) or passive (b = 0.01, SE = 0.04, *p* = .699, *β* = 0.01) sexting. The same was true with regard to the interaction term perceived susceptibility to peer-pressure × parental love and support, which was not significant for either active (b = 0.02, SE = 0.04, *p* = .681, *β* = 0.01) or passive (b = 0.05, SE = 0.05, *p* = .323, *β* = 0.02) sexting.

## Discussion

Sexting is a relatively common experience for young people and is associated with both positive and negative outcomes (e.g., Medrano et al., 2018; Mitchell et al., 2012). To our surprise, efforts to understand these experiences and behaviors using multiple-level variables drawn from different areas of the social-ecological framework (Bronfenbrenner, 1979) are absent from the research literature. This study extends previous work by considering whether school, family, and interpersonal variables (microsystem), and specific interactions between these (mesosystem) can help researchers and practitioners to better understand both active sexting behaviors and passive sexting experiences during adolescence. In our main analyses, there was no evidence that school- or family-level variables interacted with perceptions of susceptibility to peer- and/or romantic-pressure in reports of sexting behavior. There were main effects of parental love and support on both active and passive sexting, and the same for perceived susceptibility to romantic-pressure. However, there were no unique effects of perceived susceptibility to peer-pressure, and school connectedness was associated with passive, but not active sexting. Finally, young people who were attending schools engaging with the MVP intervention reported higher rates of active sexting. Exploratory analyses revealed that perceived susceptibility to peer-pressure was significant when perceived susceptibility to romantic-pressure was omitted from the statistical models, highlighting its importance outside of romantic relationships.

School connectedness was significantly and negatively associated with passive sexting but was not associated with active sexting. Using school-based interventions (Centers for Disease Control and Prevention, 2009; King et al., 2002), school connectedness can be improved, and the results presented here suggest that such interventions may help reduce the extent to which young people are asked to send sexts and have unsolicited sexts sent to them. However, our results provide less support for the possibility that improvements in school connectedness could reduce more active sexting behaviors (e.g., sending sexts).

The second school-level factor, whether young people were attending MVP schools or not, was associated with active sexting but not with passive sexting. Contrary to what was expected, young people in MVP schools reported a higher prevalence of active sexting than those not in MVP schools. No previous studies have specifically examined sexting in the context of the MVP intervention, and it is important to note that the nature of the present study precludes the interpretation that the intervention increases such behavior. For example, it may be that young people in MVP schools engaged in more sexting prior to taking part in the intervention. A more complex research design (e.g., a randomized controlled trial) is required to begin to untangle such effects. For now, though, it is interesting to note that the two school-level factors assessed in this study were associated with different forms of sexting. This may be evidence that it is important to carefully consider the nature of any intervention when seeking to influence different forms of sexting.

There were interesting differences concerning young people’s perceived susceptibility to different forms of pressure, and in how these related to their reports of sexting. There were significant, positive bivariate correlations between both forms of sexting and both peer- and romantic-pressure, but in the main analyses, the effect of perceived peer-pressure was not significant. This suggests that any association of sexting with general peer-pressure may be unimportant compared to the pressure within a romantic relationship. At the same time, the results of our exploratory analyses, which did not include perceived susceptibility to romantic-pressure, remind us of the importance of considering peer-pressure more generally, especially since not all sexting will necessarily take place within a romantic relationship. During adolescence, young people learn how to navigate romantic interactions, and this can involve learning about issues such as showing respect, making sacrifices, being assertive, and building trust (Dmytro et al., 2013). Additionally, the digital world is another contextual condition that young people need to consider (Cameron et al., 2017). The current findings suggest that learning how to cope with romantic-pressures surrounding sexting may be a salient part of this digital contextualization of relationships and their progression. Helping young people to make informed and responsible choices about sharing intensely personal thoughts, opinions, or images should therefore include ways in which they can effectively and sensitively manage and respond to partners’ sexting advances.

Finally, parental love and support was negatively related to both active and passive sexting. This extends previous research by looking at young people’s reports of both engaging in, and experiencing sexting. Importantly, this highlights the important role that families can play in shaping the ways in which young people use technology in romantic relationships. It is notable here that the items employed in our study to assess parental love and support reflected the presence of clear rules, encouragement, and expressing interest and support in their future plans. This form of supportive parenting may encourage young people to take control of their own lives and to be more assertive when sexting arises in interpersonal relationships. Notably, this type of parenting may contrast sharply with parenting that is over-bearing or perceived to be intrusive as this latter parenting (in the form of monitoring phones for sexting activity) seems to be unrelated to levels of sexting (Martinez-Prather & Vandiver, 2014). A qualitative approach to understanding the role of families in sexting could helpfully explore such issues.

Overall, there was only partial support for Hypothesis 1a. With regard to Hypothesis 1b, in which school- and family-level variables were hypothesized to interact with peer-pressure variables, there was no support, as no interactions were significant (even in the context of our relatively large sample size). This argues against the notion of a mesosystem (interaction between microsystems) operating with respect to sexting, at least with regard to the variables and interactions assessed here. This has implications for intervention strategies because it suggests that the effects of both school connection and of parental love and support are independent of perceived romantic-pressure. In such a context, it is important to develop multi-stranded interventions that aim to address multiple factors associated with sexting: romantic- and peer-pressure, school connectedness, and parental love and support.

This study has a number of strengths, including the large sample size, examination of the multiple microsystem factors simultaneously, and the investigation of the mesosystem. However, it is important to also note that the cross-sectional design of the current study precludes any causal inferences concerning the impact of microsystem variables on sexting. Thus, for example, the MVP program may be associated with higher levels of active sexting because such differences may partly drive schools to enlist in a gender-based violence intervention, to begin with. The most rigorous way to assess the impact of the intervention is to use pre- and post-assessments, with control schools, and to randomly allocate all schools to control or treatment conditions, but this was outside the scope and remit of the current study. The reliance on self-report measures may also be a weakness of the current study since it potentially leads to shared-method variance, though the extent to which this is a problem is contentious and different variables and measures can impact how much variance is shared (Richardson et al., 2009).

Past research has varied in whether reports of sexting differed according to young people’s sex. Among the young people in the current study, girls reported more involvement in passive sexting, but there was no difference between boys and girls in terms of active sexting. Taken together with the mixed reports in the research literature concerning any such difference and the direction of it (Lippman & Campbell, 2014; Marcum et al., 2014; Martinez-Prather & Vandiver, 2014; Mitchell et al., 2012; Morelli et al., 2016; Strassberg et al., 2013; Van Ouytsel et al., 2014), this may suggest that it is important to consider the form that sexting takes if seeking to understand and intervene in episodes of adolescent sexting. The nature of the items we used to assess passive sexting, combined with our results concerning perceived susceptibility to romantic-pressure, suggests that it may be particularly important to support girls and young women to be comfortable and confident should they wish to rebuff unwanted requests for sexts (or indeed unwanted sexts) from their romantic partners.

### Conclusions

This study is the first to examine the associations of sexting experiences and behaviors with multiple variables drawn from the social-ecological framework (Bronfenbrenner, 1979). The importance of both school- and family-level factors in the context of young people’s sexting interactions was evident, though perceived romantic-pressure had the largest effect. Neither school- nor family-level variables reduced the effect associated with perceived romantic-pressure. Future intervention efforts should primarily seek to tackle young people’s ability to respond effectively to romantic-pressure within romantic relationships and more general peer-pressure outside of these. They may also benefit from efforts to encourage school connectedness and to help families provide the support that is constructive and not intrusive.

## Footnotes

1 Our preregistration on the Open Science Framework includes hypotheses, copies of all measures, relevant data, Mplus code, and other project materials such as participant consent and information sheets.
